# Evaluation of QIAreach QuantiFERON-TB lateral-flow nanoparticle fluorescence assay for TB infection diagnosis among TB household contacts in three high-burden settings

**DOI:** 10.1371/journal.pone.0332125

**Published:** 2025-09-19

**Authors:** Thobani Ntshiqa, Akinlolu Fasanmi, Jeniffer Nagudi, Lindiwe Tsope, Andrew Copas, Stacie Stender, Issa Sabi, Elias Nyanda Ntinginya, Julieth Lalashowi, Manthomeng Matete, Lula Budiaki, Malebo Mahlalela, Abisai Kisinda, Don Mudzengi, Lilian Tina Minja, Tobias Chirwa, Knut Lönnroth, Stefan Niemann, Viola Dreyer, Salome Charalambous, Kavindhran Velen, Yohhei Hamada, Molebogeng Rangaka

**Affiliations:** 1 The Aurum Institute, Johannesburg, South Africa; 2 School of Public Health, University of the Witwatersrand, Johannesburg, South Africa; 3 Institute for Global Health, University College London, United Kingdom; 4 Jhpiego, Baltimore, Maryland, United States of America; 5 Mbeya Medical Research Centre, National Institute for Medical Research, Tanzania; 6 Jhpiego, Monitoring, Evaluation and Research Division, Maseru, Lesotho; 7 Karolinska Institutet, Stockholm, Sweden; 8 Molecular and Experimental Mycobacteriology, Research Center Borstel Leibniz Lung Center, Borstel, Germany; 9 German Centre for Infection Research (DZIF), Partner site Hamburg-Lübeck-Borstel-Riems, Borstel, Germany; The University of Georgia, UNITED STATES OF AMERICA

## Abstract

**Background:**

Tuberculosis infection (TBI) testing, and treatment are fundamental to achieve TB elimination. TBI testing among close or household contacts (HHCs) has been limited, in part due to perceived complexity and high operational cost. We evaluated the performance of a new near-patient and field-based QIAreach QuantiFERON-TB (QIAreach) against QuantiFERON-TB-Gold-Plus (QFT-Plus) among HHCs of people with TB.

**Methods:**

A cross-sectional study was conducted from July 2021 to September 2022 in Lesotho, South Africa and Tanzania. Blood samples were collected from HHCs for paired QFT-Plus and QIAreach processing, testing and interpretation. To evaluate the performance of QIAreach against QFT-Plus as a reference, we determined the: i) prevalence of TBI, ii) total concordance using Cohen’s Kappa, iii) predictors of discordant results using logistic regression, and iv) relationship between time to results and interferon-gamma (IFN-γ) response levels using linear correlation.

**Results:**

Out of 964 enrolled HHCs, 464 had paired results, of whom 64.9% (302/465) were female with a cohort median age of 27 years (interquartile range (IQR): 13–45). Overall, 50.9% (236/464) tested positive on QFT-Plus, while 57.1% (265/464) were positive on QIAreach assay. Total concordance between QFT-Plus and QIAreach was 78.4% [353/450, 95% confidence interval (CI): 74.4–82.2, Cohen’s Kappa: 0.5627, p < 0.001]. Discordance between assays was 23.9% (111/464) and was associated with Lesotho site (adjusted odds ratio 2.70, 95%CI: 1.48–4.92, p = 0.001). HHCs with higher IFN-γ response (QFT-Plus) (≥0.35 IU.ml-^l^) had a shorter time to results on QIAreach. In addition, a strong negative correlation between QIAreach time to results and IFN-γ response (QFT-Plus) levels (R = −0.64, 95% CI: −0.87 to −0.41, p < 0.001) was observed.

**Conclusion:**

QIAreach demonstrated a moderate concordance against QFT-Plus among HHCs in three high-burden countries. Further work is needed to understand and improve its usability in high TB and low resource settings.

## Introduction

Tuberculosis (TB), caused by *Mycobacterium tuberculosis* (*Mtb*), is a leading cause of mortality worldwide from a single infectious agent [1]. TB infection (TBI) remains a major reservoir of the TB burden which accounts for approximately 23% of the world’s population [2–4]. TBI is typically characterised by a positive tuberculin skin test (TST), interferon-gamma (IFN-γ) release assay (IGRA), or *Mtb* antigen-based skin tests (TBSTs) [5–8].

Although there is currently no gold standard for TBI diagnosis, TST is the most widely used diagnostic tool but it has limitations such as delayed immune reaction post-exposure, requires two visits, poor specificity, cross-reactivity with Bacillus Calmette–Guérin (BCG), boosting and false negativity due to anergy [9]. Other operational challenges associated with TST include lack of quality control, need for cold chain, wastage, subjective measurement of test results (prone to intra- and inter-reader variations), and challenges in data recording, training needed for standardized administration and reading of results [10–12]. Newer skin tests that are highly specific to TB have recently been approved by World Health Organisation for diagnosis of TBI. However, they still need two clinical visits for administration and reading at follow-up.

IGRAs are *in vitro* blood and enzyme-linked immunosorbent based assays (ELISA), which have been developed to overcome some of TST challenges. These assays typically contain a mixture of synthetic peptides from three *Mtb* antigens including 6-kDa early secreted antigenic target (ESAT-6), 10-kDa culture filtrate protein (CFP-10) and Rv2654 antigen (TB7.7) [6,13–15]. QuantiFERON-TB-Gold-Plus (QFT-Plus) is the fourth generation of IGRA, contains peptides from only the ESAT-6 and CFP-10 antigens and has an additional antigen tube (TB1 and TB2) [6,13–15]. TB2 tube contains both long and short ESAT-6 and CFP-10 peptides, designed to elicit IFN-γ release from both CD4 + helper T lymphocytes and CD8 + cytotoxic T lymphocytes [6,13–16]. However, the uptake of IGRAs is often limited due to logistical difficulties associated with implementing IGRA. They need a complex specimen handling and sophisticated laboratory requirements, multi-steps, time consuming tests, infrastructure, highly skilled personnel, quality support and high direct costs [17–21]. This highlights the urgent need for more field-friendly or point-of-care diagnostic tests.

Lateral flow immunoassays are portable, easy to use outside specialized laboratory environments, and provide a quick readout, making them ideal near point-of-care tests [17,22,23]. In 2021, Qiagen developed a new diagnostic test for TBI, QIAreach QuantiFERON-TB (QIAreach), a lateral-flow-nanoparticle-fluorescence assay. QIAreach, which uses the same test tube as the TB2 tube of QFT-Plus, is an easy-to-use test requiring no ELISA, less instrumentation and blood volume than QFT-Plus [17,22,23]. However, QIAreach still needs 1 ml of venous blood, incubator for 16–24 hours, with or without the centrifuge for plasma separation. Currently, limited data exists comparing its performance to QFT-Plus in high TB burdened settings. To evaluate the performance QIAreach against QFT-Plus as a reference, we determined the: i) prevalence of TBI, ii) total concordance, iii) predictors of discordant results, and iv) relationship between QIAreach time to results and IFN-γ response (QFT-Plus) levels among household contacts (HHCs) of people with TB (PWTB) in Lesotho, South Africa, and Tanzania.

## Materials and methods

### Study design

We conducted a cross-sectional study as part of a larger project titled “Community and Universal Testing for TB among contacts (CUT-TB).” CUT-TB was a pragmatic cluster-randomised trial (ISRCTN10003903) conducted in two phases in Lesotho, South Africa, and Tanzania [24]. Phase I was conducted from July 2021 to September 2022 as a pilot in preparation for the delivery of a pragmatic cluster-randomised trial. The site in South Africa received approvals from Wits Human Research Ethics Committee (9 March 2021) and Ekurhuleni District Ethics Committee (14 May 2021). The site started enrolments on the 6^th^ of July 2021 and completed on the 24^th^ of August 2022. In Lesotho, enrolment activities started on the 15^th^ of December 2021 following receipt of ethical clearance from Lesotho Ministry of Health Research and Ethics Committee (15 November 2021) and Johns Hopkins Bloomberg School of Public Health’s Institutional Review Board (9 December 2021). The site completed enrolment activities on the 3^rd^ of August 2022. Tanzania site started enrolment activities on the 17^th^ of December 2021 following receipt of ethical clearance from Mbeya Medical Research and Ethics Review Committee (9 November 2021) and Tanzania Medical Research Coordinating Committee (7 October 2021). Enrolment activities were completed on the 31^st^ of August 2022 with few follow ups in September 2022.

### Study setting

Data collection was conducted in the peri-urban communities within three Sub-Saharan countries, including Lesotho (Maseru, Thaba-Tseka, and Quthing districts), South Africa (Ekurhuleni district), and Tanzania (Songwe and Mbeya regions). The chosen study sites were situated in regions with varying background TB incidence rates, socio-economic profiles, and differing HIV prevalence. Testing was done in Lesotho’s National TB Reference Laboratory, Aurum’s Tembisa Clinical Research laboratory for South Africa and NIMR – Mbeya Medical Research Center laboratory for Tanzania site.

### Study population

The study population were HHCs of people aged ≥18 years with microbiologically-confirmed drug-sensitive and/or drug-resistant TB. We identified PWTB who were diagnosed within six weeks of being approached by study team from health facilities within the selected districts or regions. HHCs listed by each TB index patient were enumerated and recruited during household visits to participate in the study. A HHC was defined as any person who shared the same enclosed living space for seven nights or for frequent or extended periods during the day with the index patient during the three months before commencement of the current treatment episode. For the present study, blood samples were collected only from HHCs aged ≥5 years.

### Blood collection, processing and testing

A 6 ml of whole blood sample was collected intravenously from each participant into a single lithium heparin tube for QFT-Plus [25,26] and QIAreach (17,23) processing, testing and interpretation following manufacturer’s guidelines. Samples were then transported to the laboratory where they were aliquoted into 1 ml tubes for QFT-Plus nil, QFT-Plus TB1, QFT-Plus TB2, QFT-Plus mitogen, and QIAreach tube. Tubes were then placed in a pre-heated 37°C portable incubator for 16–24 hours, within 8 hours of collection. Subsequently, samples were centrifuged to separate plasma for same day testing. Alternatively, plasma was transferred into a microtiter tube and stored between −20°C to −80°C until testing, mostly within 48–72 hours.

The manufacturer (Qiagen) conducted onsite lab training for at least two lab personnel in each site before QIAreach testing. Few samples were tested while the product specialist was onsite as part of training and quality assurance. Before testing, QIAreach Software was installed into a laptop with Microsoft Windows system. Before it was connected to the computer via a USB cable, an eHub was fully charged for few hours. The eStick was then inserted into the eHub port after it was powered and turned on. After the computer system was in a ready mode, we added 150 *µ*L of diluent buffer into a processing tube. We then transferred 150 *µ*L of plasma specimen into the same processing tube. This was then mixed four to six times using a pipet before the mixture was aliquoted from the processing tube into the sample port of the inserted eStick. Upon sensing 150 *µ*L of the mixture in the eStick, the eHub and the computer system automatically displayed the status of the assay as pending. After completion, both the eHub and the QIAreach Software displayed the final results as either positive or negative and as well as time to result.

### Study outcome

This analysis focused on two main study outcomes: i) the proportion of participants who had a positive QFT-Plus results, herein referred to as TBI prevalence, and ii) the proportion of QFT-Plus results which agree with QIAreach results, herein referred to as concordance. Although not a gold standard, QFT-Plus was used as a reference standard to determine TBI prevalence. Indeterminate QFT-Plus results were not included in the concordance calculation.

### Statistical methods

We assessed concordance between QFT-Plus and QIAreach results, and percentage agreements were summarised using overall percent agreement (OPA), positive percent agreement (PPA; sensitivity) and negative percent agreement (NPA; specificity) with their corresponding 95% Confidence Intervals (CI) and p-values using QFT-Plus as a reference. Cohen’s Kappa statistics was used to assess total concordance. Logistic regression was used to determine predictors of discordance between QFT-Plus and QIAreach. Mann-Whitney U (Wilcoxon rank sum) and linear correlation were used to assess the relationship between QIAreach time to results and quantitative QFT-Plus IFN-γ response levels. A p ≤ 0.05 was considered significant for all statistical tests carried out. Data was analysed using Stata version 16 (StataCorp. 2017. Stata Statistical Software: Release 16. College Station, TX: StataCorp LP).

### Ethical considerations

The study received ethical clearance from Wits Human Research Ethics Committee (210107), Ekurhuleni District Health Ethics Committee (GP_202104_015) in South Africa; Johns Hopkins Bloomberg School of Public Health’s Institutional Review Board (16967) and Lesotho National Health Research Ethics Committee (37–2021) in Lesotho; and Tanzania Medical Research Coordinating Committee (NIMR/HQ/R.8a/Vol.IX/3799) and Mbeya Medical Research and Ethics Review Committee (SZEC-2439/R.C/V.1/55). Permissions were also sought from districts and other external stakeholders. Prior to commencement of study procedures, we sought informed consent from all study participants using written informed consent and information sheet available in the commonly used local languages. An impartial witness was used to witness the verbal consent for illiterate participants. A thumb print was used to document or “sign” a verbal consent.

### Inclusivity in global research

Additional information regarding the ethical, cultural, and scientific considerations specific to inclusivity in global research is included in the Supporting Information (S1 File).

## Results

### Participants enrolment

**Fig 1:**
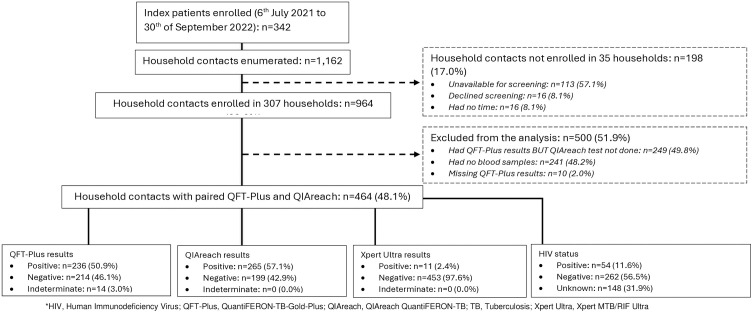
Participants flow chart at enrolment for TB index patients and their household contacts in three high TB burden countries.

Between the 6^th^ of July 2021 and 30^th^ of September 2022, a total of 342 PWTB were enrolled: 100 (29.2%) Lesotho, 152 (44.4%) South Africa and 90 (26.3%) in Tanzania. In total, 1,162 HHCs were enumerated of whom 83.0% (964/1,162) were enrolled from 307 (89.8%) households. Of the 964 enrolled HHCs, only 464 had paired QFT-Plus and QIAreach results due to limited QIAreach kit supply. All tested participants were aged ≥5 years, 2.4% (11/464) had positive Xpert Ultra, and 11.6% (54/464) were living with HIV (Fig 1).

### Demographic characteristics of participants

Overall, 64.9% (301/464) of the enrolled HHCs were females and the median age was 27 years (IQR: 13–45). Of this, 47.8% (222/464) were from Lesotho, 26.3% (122/464) from South Africa, while 25.9% (120/464) were from Tanzania (Table 1).

**Table 1:** Demographic characteristics of household contacts (N = 464) in three high TB burden countries.

Variable (N = 464)		All – n (%)	QFT-Plus			p-Value	QIAreach		p-Value	Difference in TBI detection
			Positive – n (%)	Negative – n (%)	Indeterminate – n (%)		Positive – n (%)	Negative – n (%)		
Age, years		464 (100.0%)	236 (50.9%)	214 (46.1%)	14 (3.0%)	<0.001	265 (57.1%)	199 (42.9%)	<0.001	29
	Median (IQR)	27 (13, 45)	32 (18, 52)	18.5 (11, 37)	21.5 (10, 53)		32 (18, 52)	17 (11, 34)		
Age group						<0.001			<0.001	
	<10 years	58 (12.5%)	24 (41.4%)	31 (53.5%)	3 (5.2%)		22 (37.9%)	36 (62.1%)		−2
	10-19 years	129 (27.8%)	45 (34.9%)	82 (63.6%)	2 (1.6%)		53 (41.1%)	76 (58.9%)		8
	20-29 years	67 (14.4%)	37 (55.2%)	27 (40.3%)	3 (4.5%)		39 (58.2%)	28 (41.8%)		2
	30-39 years	67 (14.4%)	40 (59.7%)	27 (40.3%)	0 (0.0%)		47 (70.2%)	20 (29.9%)		7
	40-49 years	46 (9.9%)	28 (60.9%)	17 (37.0%)	1 (2.2%)		30 (65.2%)	16 (34.8%)		2
	≥50 years	97 (20.9%)	62 (63.9%)	30 (30.9%)	5 (5.2%)		74 (76.3%)	23 (23.7%)		12
Gender						0.461			0.246	
	Female	301 (64.9%)	147 (48.8%)	145 (48.2%)	9 (3.0%)		166 (55.2%)	135 (44.9%)		19
	Male	163 (35.1%)	89 (54.6%)	69 (42.3%)	5 (3.1%)		99 (60.7%)	64 (39.3%)		10
Country						0.009			0.004	
	Lesotho	222 (47.8%)	121 (54.5%)	93 (41.9%)	8 (3.6%)		120 (54.1%)	102 (45.9%)		−1
	South Africa	122 (26.3%)	64 (52.5%)	52 (42.6%)	6 (4.9%)		85 (69.7%)	37 (30.3%)		21
	Tanzania	120 (25.9%)	51 (42.5%)	69 (57.5%)	0 (0.0%)		60 (50.0%)	60 (50.0%)		9
HIV status						0.017			0.038	
	Negative	262 (56.5%)	148 (56.5%)	107 (40.8%)	7 (2.7%)		163 (62.2%)	99 (37.8%)		15
	Positive	54 (11.6%)	28 (51.9%)	26 (48.2%)	0 (0.0%)		26 (48.2%)	28 (51.9%)		−2
	Unknown	148 (31.9%)	60 (40.5%)	81 (54.7%)	7 (4.7%)		76 (51.4%)	72 (48.7%)		16
Xpert Ultra results						0.036			0.082	
	Negative	453 (97.6%)	230 (50.8%)	211 (46.6%)	12 (2.7%)		256 (56.5%)	197 (43.5%)		26
	Positive	11 (2.4%)	6 (54.6%)	3 (27.3%)	2 (18.2%)		9 (81.8%)	2 (18.2%)		3

*HIV, Human Immunodeficiency Virus; IQR, Interquartile Range; QFT-Plus, QuantiFERON-TB-Gold-Plus; QIAreach, QIAreach QuantiFERON-TB; TB, Tuberculosis; Xpert Ultra, Xpert MTB/RIF Ultra

### Prevalence of TB infection

Overall, 50.9% (236/464) of enrolled HHCs tested positive on QFT-Plus, while 57.1% (265/464) were positive on QIAreach assay. The median age of HHCs with positive QFT-Plus and/or QIAreach results [32 years (IQR: 18–52), p < 0.001] was higher compared to median age of HHCs with negative QFT-Plus and QIAreach results. Test positivity increased with increasing age in both assays, with QIAreach identifying more cases in those aged ≥50 years [19,4% (12/62)]. Overall, QIAreach showed a higher positivity among HHCs in South Africa [69.7% (85/122)] and among those with Xpert Ultra positive results [81.8% (9/11)] compared to QFT-Plus (Table 1).

### Concordance of QFT-Plus vs QIAreach

Of the HHCs with paired tests, 42.5% (197/464) were positive and 33.6% (156/464) were negative on both assays. On the other hand, 23.9% (111/464) had discordant results: 35.1% (39/111) QFT-Plus positive/QIAreach negative, 52.3% (58/111) QFT-Plus negative/QIAreach positive, 9.0% (10/111) QFT-Plus indeterminate/QIAreach positive, and 3.6% (4/111) QFT-Plus indeterminate/QIAreach negative. Overall, the concordance (also known as OPA) between QFT-Plus and QIAreach was 78.4% [353/450, 95% CI: 74.4–82.2, Cohen’s Kappa: 0.5627, p < 0.001] with a PPA of 83.5% (197/236, 95% CI: 78.1–88.0) and NPA of 72.9% (156/214, 95% CI: 66.4–78.7). Discordance between QFT-Plus and QIAreach was higher in Lesotho [28.5% (61/214), 95% CI: 22.6–35.1) compared to Tanzania [14.2% (17/120), 95% CI: 8.5–21.7] and South Africa [16.4% (19/116), 95% CI: 10.2–24.4] (Table 2).

Overall, the proportion of HHCs with discordant results was 23.9% (111/464). On multivariable analysis, having discordant results was significantly associated with being from Lesotho (aOR 2.70, 95% CI: 1.48–4.92, p = 0.001) (Table 3).

**Table 2:** Diagnostic performance of QIAreach vs QFT-Plus among household contacts in three high TB burden countries (N = 464).

QFT-Plus	N	QIAReach			Concordance and Agreement	
Positive	Negative	Indeterminate	Concordance	78.4% (353/450); 95% CI: 74.4–82.2; k = 0.5661; p < 0.001
Positive	236	197	39	0	Discordance	21.6% (97/450); 95% CI: 17.8–25.6
Negative	214	58	156	0	Overall percentage agreement	78.4% (353/450); 95% CI: 74.4–82.2
Indeterminate	14	10	4	0	Positive percentage agreement^	83.5% (197/236); 95% CI: 78.1–88.0
Total	464	265	199	0	Negative percentage agreement^	72.9% (156/214); 95% CI: 66.4–78.7
**Participants who have QFT-Plus and QIAReach for Lesotho**						
**QFT-Plus**	**N**	**QIAReach**			**Concordance and Agreement**	
		Positive	Negative	Indeterminate	Concordance	71.5% (153/214); 95% CI: 64.9–77.4; k = 0.4236; p < 0.001
Positive	121	88	33	0	Discordance	28.5% (61/214); 95% CI: 22.6–35.1
Negative	93	28	65	0	Overall percentage agreement	71.5% (153/214); 95% CI: 64.9–77.4
Indeterminate	8	4	4	0	Positive percentage agreement^	72.7% (88/121); 95% CI: 63.9–80.4
Total	222	120	102	0	Negative percentage agreement^	69.9% (65/93); 95% CI: 59.5–79.0
**Participants who have QFT-Plus and QIAReach for South Africa**						
**QFT-Plus**	**N**	**QIAReach**			**Concordance and Agreement**	
		Positive	Negative	Indeterminate	Concordance	83.6% (97/116); 95% CI: 75.6–89.8; k = 0.6597; p < 0.001
Positive	64	62	2	0	Discordance	16.4% (19/116); 95% CI: 10.2–24.4
Negative	52	17	35	0	Overall percentage agreement	83.6% (97/116); 95% CI: 75.6–89.8
Indeterminate	6	6	0	0	Positive percentage agreement^	96.9% (62/64); 95% CI: 89.2–99.6
Total	122	85	37	0	Negative percentage agreement^	67.3% (35/52); 95% CI: 52.9–79.7
**Participants who have QFT-Plus and QIAReach for Tanzania**						
**QFT-Plus**	**N**	**QIAReach**			**Concordance and Agreement**	
		Positive	Negative	Indeterminate	Concordance	85.8% (103/120); 95% CI: 78.3–91.5; k = 0.7167; p < 0.001
Positive	51	47	4	0	Discordance	14.2% (17/120); 95% CI: 8.5–21.7
Negative	69	13	56	0	Overall percentage agreement	85.8% (103/120); 95% CI: 78.3–91.5
Indeterminate	0	0	0	0	Positive percentage agreement^	92.2% (47/51); 95% CI: 81.1–97.8
Total	120	60	60	0	Negative percentage agreement^	81.2% (56/69); 95% CI: 69.9–89.6

^QFT-Plus considered an imperfect reference standard.

*CI, Confidence Interval; QFT-Plus, QuantiFERON-TB-Gold-Plus; QIAreach, QIAreach QuantiFERON-TB; TB, Tuberculosis

**Table 3:** Factors associated with discordant QFT-Plus vs QIAreach results among household contacts in three high TB burden countries (N = 464).

Variable (N = 464)		All	Discordant QFT-Plus vs QIAreach - n (%)	Univariate analysis			Multivariable analysis		
				Crude OR	95% CI	p-Value	Adjusted OR	95% CI	p-Value
Age group		464	111 (23.9%)						
	<10 years	58	13 (22.4%)	1	Reference	Reference	1	Reference	Reference
	10-19 years	129	29 (22.5%)	1.00	0.48–2.11	0.992	0.89	0.42–1.91	0.766
	20-29 years	67	22 (32.8%)	1.69	0.76–3.77	0.198	1.38	0.60–3.16	0.443
	30-39 years	67	17 (25.4%)	1.18	0.51–2.69	0.699	1.07	0.46–2.52	0.871
	40-49 years	46	8 (17.4%)	0.73	0.27–1.94	0.527	0.69	0.25–1.91	0.479
	≥50 years	97	22 (22.7%)	1.02	0.47–2.21	0.969	0.89	0.39–2.01	0.777
Gender									
	Female	301	67 (22.3%)	1	Reference	Reference	1	Reference	Reference
	Male	163	44 (27.0%)	1.29	0.83–2.00	0.254	1.25	0.79–1.97	0.348
Country									
	Tanzania	120	17 (14.2%)	1	Reference	Reference	1	Reference	Reference
	South Africa	122	25 (20.5%)	1.56	0.79–3.07	0.196	1.54	0.77–3.10	0.224
	Lesotho	222	69 (31.1%)	2.73	1.52–4.91	0.001	2.70	1.48–4.92	0.001
HIV status									
	Negative	262	59 (22.5%)	1	Reference	Reference	–	–	–
	Positive	54	12 (22.2%)	0.98	0.49–1.99	0.962	–	–	–
	Unknown	148	40 (27.0%)	1.27	0.80–2.03	0.306	–	–	–
Xpert Ultra results									
	Negative	453	108 (23.8%)	1	Reference	Reference	–	–	–
	Positive	11	3 (27.3%)	1.20	0.31–4.60	0.792	–	–	–

*CI, Confidence Interval; HIV, Human Immunodeficiency Virus; OR, Odds Ratio; QFT-Plus, QuantiFERON-TB-Gold-Plus; QIAreach, QIAreach QuantiFERON-TB; TB, Tuberculosis; Xpert Ultra, Xpert MTB/RIF Ultra.

### Relationship between QIAreach time to results and QFT-Plus IFN-γ response levels

**Fig 2:**
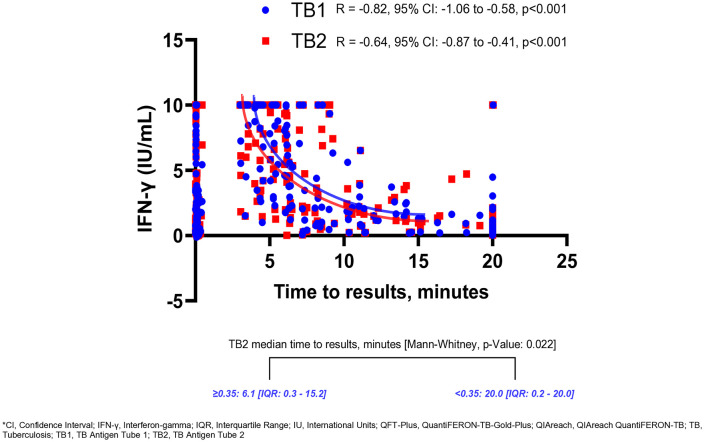
QIAreach time to result correlation with QFT-Plus among household contacts in three high TB burden countries.

TB2 had a higher median IFN-γ response (QFT-Plus) [0.51 IU^.^ml^-l^ (IQR: 0.12–3.75)] compared to TB1 [0.46 IU^.^ml^-l^ (IQR: 0.10–2.86)]. In addition, the proportion of HHCs with higher IFN-γ response (QFT-Plus) (≥0.35 IU^.^ml^-l^) was 55.8% (259/464) in TB2. Overall, the median time to results was 6.2 minutes (IQR: 0.2–20.0) for QIAreach. However, those with higher IFN-γ response (QFT-Plus) (≥0.35 IU^.^ml^-l^) had a shorter QIAreach time to results [6.1 minutes (IQR: 0.2–15.2), p = 0.022] compared to those who had a lower IFN-γ response (QFT-Plus) (<0.35 IU^.^ml^-l^). On linear correlation, TB1 demonstrated a stronger negative correlation between QIAreach time to results and IFN-γ response (QFT-Plus) levels (correlation coefficient R = −0.82, 95% CI: −1.06 to −0.58, p < 0.001) compared to TB2 (correlation coefficient R = −0.64, 95% CI: −0.87 to −0.41, p < 0.001) (Fig 2).

## Discussion

In this study, we evaluated the performance of QIAreach for TBI testing. To do this, we determined the prevalence of TBI among HHCs of PWTB using QFT-Plus as reference in three high TB burden countries. We found that more than half of HHCs had TBI. We also evaluated concordance between the two assays and found that QIAreach had a moderate concordance against QFT-Plus although there was a slight variation between countries. Discordance between the two assays was associated with Lesotho site. Additionally, a strong negative correlation and/or inverse relationship between time to results and IFN-γ response (QFT-Plus) levels was observed. Our data contributes to existing evidence on diagnostic performance of QIAreach against QFT-Plus. It is also the first performance evaluation to be conducted in high TB incidence setting and outside of the health facility setting to facilitate access to IGRA closer to the point-of-care. However, QIAreach testing still requires skilled personnel to draw blood.

Using QFT-Plus as a reference, TBI prevalence found in this study is consistent with estimates from systematic reviews which ranged between 42.4% to 51.5% among TB exposed contacts in high, middle, and low income countries [27–29]. QIAreach identified 29 additional HHCs with TBI, with those from South Africa and those with Xpert Ultra positive results more likely to test positive on QIAreach compared to QFT-Plus. Similar to findings from a trial conducted in Vietnam, QIAreach (45.6%, 119/261) had a higher positivity compared to QFT-Plus (25.3%, 66/261) and identified 59 additional contacts with TBI [18]. Similar findings were also observed in a recent study in Malawi [30], where QIAreach showed more than two times higher positivity rate compared to QFT-Plus with low concordance (Cohen’s Kappa: 0.26, 95% CI: 0.20–0.32). In contrast, other studies conducted in low TB settings found similar positivity between the two assays [11,17,21,23]. Although QIAreach demonstrated a higher number of people with TBI compared to QFT-Plus in our study, absence of a definitive gold standard for diagnosis of TBI complicates the interpretation of discordant results between these two assays [30,31]. Thus, it remains unclear if these additional HHCs with TBI are at an increased risk for TB. Higher positivity rates observed with QIAreach in this and previous studies (e.g., Malawi, Vietnam) may reflect enhanced sensitivity or immune variability among populations. Given the absence of a definitive reference standard, these findings should be interpreted cautiously and warrant prospective follow-up to determine clinical relevance.

The interpretation of discordant results is further complicated by frequency of QFT-Plus indeterminate results, which was observed in 14 (3.0%) HHCs in this study. QFT-Plus indeterminate rate found in this study is slightly lower than the pooled estimate (3.9%, 95% CI: 3.5–4.2) reported in recent systematic review [31]. Of the 14 HHCs with indeterminate QFT-Plus results, 10 were positive on QIAreach test, which contributed to a higher positivity rate of QIAreach. Previous studies either excluded indeterminate QFT-Plus results from performance analysis [11,18], or did not show head to head comparison of indeterminate QFT-Plus results with QIAreach [17,23]. Indeterminate QFT-Plus result is often caused by high IFN-γ response levels in the negative control (Nil tube) or a low response in the positive control (mitogen tube) [31,32], a phenomenon which is more common among those who are immunocompromised, HIV positive with low CD4 + cell count, and children [31], who are at risk for TB. QIAreach has one blood collection tube without negative (Nil) or positive (mitogen) controls, while QFT-Plus has four tubes (TB1, TB2, Nil, and mitogen). Consequently, QIAreach does not have indeterminate result. There is a risk that those who are at high risk for TB are not offered TPT because of indeterminate results if QFT-Plus is the only test. Although there are concerns about absence of negative and positive controls and their impact on test performance, QIAreach may still be preferrable in those who are immunocompromised, HIV positive with low CD4 + cell count, and children to avoid undertreatment, if the decision to start treatment requires a positive TBI test. However, larger prospective studies are needed to assess its performance, to predict the development of TB in these groups.

Similar to findings from a study conducted in Vietnam [18], QIAreach demonstrated a moderate concordance against QFT-Plus in this study. The OPA (78.4%) found in this study was similar to OPA found in Vietnam (78.9%) [18] but lower compared to OPA reported in Malaysia (94.9%) [11], Italy (97.7%) [21], Japan (98.8%) [23], and USA (95.6%) [17]. In addition, the PPA (83.5%) and NPA (72.9%) found in this study were also lower compared to estimates reported in Vietnam (PPA, 98.5%; NPA, 72.3%) [18], Malaysia (PPA, 96.5%; NPA, 94.2%) [11], Italy (PPA, 100%; NPA, 96.2%) [21], Japan (PPA, 100%; NPA, 97.6%) [23], and USA (PPA, 100%; NPA, 95.6%) [17]. The concordance reported in this study was also lower compared to a pooled OPA (92%, 95% CI: 83–98), pooled PPA (98%, 95% CI: 88–100%) and pooled NPA (91%, 95% CI: 81–97%) reported in a systematic review which was conducted in low to intermediate TB settings [30]. The observed difference in performance between QIAreach and QFT-Plus remains unclear, but is likely multifactorial. It may reflect a combination of analytical and host-related factors—including differences in immune activation thresholds, background TB burden, and logistical complexity in field implementation. Such variability is not uncommon in IGRA evaluations and warrants further investigation across pre-analytical, analytical, and post-analytical domains [33–35].This variability often results in false-positive results and increased likelihood of conversion and reversion rates in reproducibility studies [31,33–35]. Other factors include borderline IFN-γ response values near the cut-off, absence of a Nil background subtraction in the QIAreach test, sample viscosity, autoimmune disease, milky plasma, and color affecting lateral flow functionality. In particular, discordance between QFT-Plus and QIAreach was associated with Lesotho site, suggesting that test performance may be affected by site or lab specific factors [11,36–38], including but not limited to environmental, experience and training differences. Although concordance with QFT-Plus was moderate—raising the possibility of over- or undertreatment relative to the reference test—the operational simplicity of QIAreach may enable broader programmatic use, particularly in resource-limited or decentralized settings. This scalability could translate into greater overall public health impact. However, well-designed implementation studies will be crucial to assess its real-world clinical effectiveness and cost-effectiveness. Although QIAreach is no longer commercially available, insights from this evaluation remain relevant to inform future development of point-of-care TB infection tests that aim to simplify IGRA workflows for field use.

We found a significant relationship between IFN-γ response (QFT-Plus) levels and QIAreach time to results. HHCs who had higher IFN-γ response (QFT-Plus) levels had shorter QIAreach time to results compared to those who had a lower IFN-γ response (QFT-Plus). Previous studies also reported similar findings [11,17,23]. This inverse relationship also suggests that time to results may be used as a surrogate marker for IFN-γ response when using QIAreach assay [23].

### Study limitations, strengths and possible future studies

Our study had several limitations beyond those discussed above. Firstly, the absence of a definitive gold standard for diagnosis of TBI complicates the interpretation of discordant results between QFT-Plus and QIAreach assays. Secondly, the study was conducted in selected regions using convenience sampling in each country, limiting the generalisability of our findings. We also did not include children aged <5 years, pregnant women (or did not record data on pregnancy status), people living with HIV, prisoners, miners, healthcare workers and other subgroups and therefore cannot comment on them.

Some of the strengths of this study include the fact that this was a multi-country study, conducted in high TB incidence settings, and designed by a multidisciplinary team with varying scientific expertise. Although statistical power was not calculated, the sample size of this study was relatively bigger compared to previous studies. Our data contributes to the body of existing evidence on diagnostic performance of QIAreach against QFT-Plus. It is also the first performance evaluation to be conducted in high TB setting and outside of the health facility setting to facilitate access to IGRA closer to the point-of-care.

## Conclusions

Among HHCs in three high-burden countries, we identified a high TBI prevalence using QFT-Plus. QIAreach demonstrated a moderate concordance against QFT-Plus; and there was an inverse relationship between time to results and IFN-γ response (QFT-Plus) levels, suggesting that time to results may be used as a surrogate marker for IFN-γ response when using QIAreach assay. However, in the absence of a gold standard test, it is difficult to interpret the implication of these findings. Further research is needed to understand its predictive performance for TB and usability in this population, specifically if it addresses field implementation challenges associated with similar TBI tests.

## Supporting information

S1 File Inclusivity in global research. (DOCX)
